# Developing an IPF Prognostic Model and Screening for Key Genes Based on Cold Exposure-Related Genes Using Bioinformatics Approaches

**DOI:** 10.3390/biomedicines13030690

**Published:** 2025-03-11

**Authors:** Peiyao Luo, Quankuan Gu, Jianpeng Wang, Xianglin Meng, Mingyan Zhao

**Affiliations:** 1 Department of Critical Care Medicine, The First Affiliated Hospital of Harbin Medical University, Harbin 150001, China; 2 Heilongjiang Provincial Key Laboratory of Critical Care Medicine, No. 2075, Qunli Seventh Avenue, Daoli District, Harbin 150001, China

**Keywords:** idiopathic pulmonary fibrosis, cold exposure, prognostic models

## Abstract

**Background:** Cold exposure has an impact on various respiratory diseases. However, its relationship with idiopathic pulmonary fibrosis (IPF) remains to be elucidated. In this study, bioinformatics methods were utilized to explore the potential link between cold exposure and IPF. **Methods:** Cold exposure-related genes (CERGs) were identified using RNA-Seq data from mice exposed to cold versus room temperature conditions, along with cross-species orthologous gene conversion. Consensus clustering analysis was performed based on the CERGs. A prognostic model was established using univariate and multivariate risk analyses, as well as Lasso–Cox analysis. Differential analysis, WGCNA, and Lasso–Cox methods were employed to screen for signature genes. **Results:** This study identified 151 CERGs. Clustering analysis based on these CERGs revealed that IPF patients could be divided into two subgroups with differing severity levels. Significant differences were observed between these two subgroups in terms of hypoxia score, EMT score, GAP score, immune infiltration patterns, and mortality rates. A nine-gene prognostic model for IPF was established based on the CERG (AUC: 1 year: 0.81, 3 years: 0.79, 5 years: 0.91), which outperformed the GAP score (AUC: 1 year: 0.66, 3 years: 0.75, 5 years: 0.72) in prognostic accuracy. IPF patients were classified into high-risk and low-risk groups based on the RiskScore from the prognostic model, with significant differences observed between these groups in hypoxia score, EMT score, GAP score, immune infiltration patterns, and mortality rates. Ultimately, six high-risk signature genes associated with cold exposure in IPF were identified: GASK1B, HRK1, HTRA1, KCNN4, MMP9, and SPP1. **Conclusions:** This study suggests that cold exposure may be a potential environmental factor contributing to the progression of IPF. The prognostic model built upon cold exposure-related genes provides an effective tool for assessing the severity of IPF patients. Meanwhile, GASK1B, HRK1, HTRA1, KCNN4, MMP9, and SPP1 hold promise as potential biomarkers and therapeutic targets for IPF.

## 1. Background

Idiopathic pulmonary fibrosis (IPF) is a chronic, progressive lung disease characterized by persistent pulmonary scarring and a histologic pattern of usual interstitial pneumonia (UIP) that is diagnostic of the condition. This disease often leads to symptoms such as exacerbated cough and dyspnea, significantly impairing patients’ quality of life and posing a significant health threat to approximately 3 million people worldwide [1,2]. The immune system plays a central driving role in the pathophysiology of IPF [3]. Marked immune cell infiltration, including lymphocytes, macrophages, and neutrophils, is observed in the lung tissue of patients. These cells release a series of inflammatory mediators and cytokines, such as TNF-α, TGF-β, and IL-8, which further activate fibroblasts and accelerate the synthesis and deposition of extracellular matrix, thereby exacerbating the fibrotic process in the lung tissue and promoting disease progression [4,5,6,7].

Ambient temperature has a significant impact on the development of respiratory diseases [8]. Cold exposure has been identified as a key environmental factor in the acute exacerbation of various respiratory diseases in multiple studies [9,10,11,12,13]. Under cold-exposure conditions, the body triggers a series of complex physiological responses, including the activation of brown adipose tissue (BAT) to maintain body temperature stability [14]. Additionally, various immune cell populations participate in the regulation of BAT activity through complex intercellular networks [15]. Furthermore, cold exposure may disrupt the complex signaling communication network between the nervous, endocrine, and immune systems, leading to changes in the immune cell composition of the body and subsequently impairing immune function [16].

Although direct evidence linking cold exposure to the exacerbation and poor prognosis of IPF is currently lacking, studies have shown that cold exposure can aggravate pulmonary inflammatory responses. For example, cold exposure can enhance systemic inflammatory responses, increase levels of pro-inflammatory cytokines, and activate signaling pathways related to NF-κB and NLRP3 inflammasomes, thereby exacerbating lung damage [17]. Cold exposure also induces a significant increase in neutrophil infiltration in the lung tissue of mice, along with a marked elevation in the production of IFN-γ-induced pro-inflammatory cytokines such as IL-12, TNF-α, IL-17, and IL-18 [18]. Furthermore, studies have demonstrated that cold exposure can lead to changes in pulmonary immune cell populations. For instance, lung tissue biopsy results after cold exposure show increased submucosal infiltration of T lymphocytes, neutrophils, macrophages, and eosinophils, accompanied by airway structural remodeling [19]. Additionally, cold exposure results in a significant increase in the number of CD4^+^ T cells in bronchoalveolar lavage fluid [20]. These findings suggest that the impact of cold exposure on the pulmonary immune microenvironment may have a potential association with IPF.

Therefore, this study aims to delve into the potential link between cold exposure and IPF using bioinformatics methods. By identifying cold-exposure-related genes in a chronic cold-exposure mouse model, we aim to construct a prognostic prediction model for IPF. Simultaneously, based on cold-exposure-related genes, we aim to identify novel genes related to the progression of IPF, providing new ideas and approaches for the prevention and treatment of this disease.

## 2. Methods

### 2.1. Data Source and Processing

Data were retrieved from the Gene Expression Omnibus (https://www.ncbi.nlm.nih.gov/geo/ (accessed on 25 November 2024)) database at the National Center for Biotechnology Information (NCBI) in the United States. The information on the datasets is presented in Table 1. The GSE183321 dataset utilized 8-week-old male C57BL/6J mice that were housed at 10 °C for 2 weeks to simulate cold exposure. For the GSE70866 expression profile, the R package in SilicoMerging [21] was employed to merge the data, and batch effects were removed using the method proposed by Johnson WE et al. [22].

### 2.2. Differential Analysis and Functional Enrichment Analysis

Microarray data and transcriptome data were subjected to differential expression analysis using the limma package [23] (v3.40.6) and DESeq2 [24] (v1.32.0) in R, respectively. The criteria for screening were set as a fold change greater than 1.5 and a *p*-value less than 0.05. The clusterProfiler package (v3.14.3) was utilized to perform Gene Ontology (GO) and Kyoto Encyclopedia of Genes and Genomes (KEGG) enrichment analyses on the differentially expressed genes. The GSEA software (v 3.0) was obtained from the GSEA website (http://software.broadinstitute.org/gsea/index.jsp (accessed on 27 November 2024)) and used for GSEA enrichment analysis based on the MSigDB database [25,26] (http://www.gsea-msigdb.org/gsea/downloads.jsp (accessed on 27 November 2024)). Additionally, Gene Set Variation Analysis (GSVA) enrichment analysis was conducted using the GSVA package (v1.40.1) based on the MSigDB database [27].

### 2.3. Weighted Gene Co-Expression Network Analysis (WGCNA)

The data were preprocessed and outliers were removed by calculating the Median Absolute Deviation (MAD) values and applying the goodSamplesGenes function from the R package. A scale-free co-expression network was constructed, which involved calculating the Pearson correlation coefficient matrix, building a weighted adjacency matrix, transforming it into a Topological Overlap Matrix (TOM), and performing average linkage hierarchical clustering based on this to establish co-expression modules. Modules significantly associated with the disease group were selected, and core genes were screened using thresholds of |MM| > 0.5 and |GS| > 0.1 [28].

### 2.4. Protein–Protein Interaction Network (PPI) Analysis

The protein–protein interaction network analysis was conducted using the STRING database [29] (https://cn.string-db.org/ (accessed on 26 November 2024)), and visualization was performed using Cytoscape software (v3.9.1) [30].

### 2.5. IPF-Related Feature Scoring

Hypoxia and epithelial–mesenchymal transition (EMT) are crucial pathological features in the development and progression of IPF [31,32,33]. We downloaded the gene sets for the HALLMARK hypoxia and EMT pathways from the MSigDB database and applied ssGSEA to the dataset to obtain IPF-related feature scores. The GAP (Gender, Age, Physiology) score system is an important clinical tool for assessing the severity of IPF and predicting prognosis [34]. In this study, the GAP scores were provided within the dataset.

### 2.6. Immune Infiltration Analysis

Immune infiltration analysis was conducted using ImmuCellAI [35] (https://guolab.wchscu.cn/ (accessed on 30 November 2024)). ImmuCellAI can assess the abundance of 24 immune cell types in human samples, including 18 T cell subtypes and 6 other immune cell types.

### 2.7. Consensus Clustering

Cluster analysis was performed using ConsensusClusterPlus [36] using agglomerative km clustering with a 1-pearson correlation distance and resampling 80% of the samples for 10 repetitions. The optimal number of clusters was determined using the empirical cumulative distribution function plot.

### 2.8. Prognostic Model Construction and Survival Risk Analysis

Survival time, survival status, and gene expression data were integrated. The R package glmnet was used to perform regression analysis using the Lasso–Cox method to construct a prognostic risk model. The R package survival was utilized for univariate and multivariate risk analysis using the Cox method. The R package rms was employed to establish a nomogram using the Cox method. Receiver operating characteristic (ROC) analysis was conducted using the R package pROC (v1.17.0.1) to obtain the area under the curve (AUC). The survfit function from the R package survival was used to analyze prognostic differences between the two groups, and the log-rank test was applied to assess the significance of prognostic differences between different sample groups.

### 2.9. Statistical Analysis

Data were analyzed using R software (v4.4.1) and the SangerBox 3.0 [37] online platform. The Wilcoxon rank-sum test and t-test were employed to assess differences between groups. A *p*-value < 0.05 was considered statistically significant.

## 3. Results

### 3.1. Identification and Clustering Analysis of Cold Exposure-Related Genes

Initially, we conducted differential analysis using the GSE183321 dataset, identifying 171 differentially expressed genes between the cold-exposure group and the control group. These genes are closely related to the function, activation, and differentiation of immune cells (Supplementary Figure S1), consistent with previous findings [38]. Since this dataset comprises samples from C57BL/6J mice, we performed cross-species ortholog conversion for the 171 genes to facilitate subsequent analyses, excluding genes without human orthologs. Ultimately, 151 human genes were identified and designated as cold exposure-related genes (CERGs).

**Figure 1:**
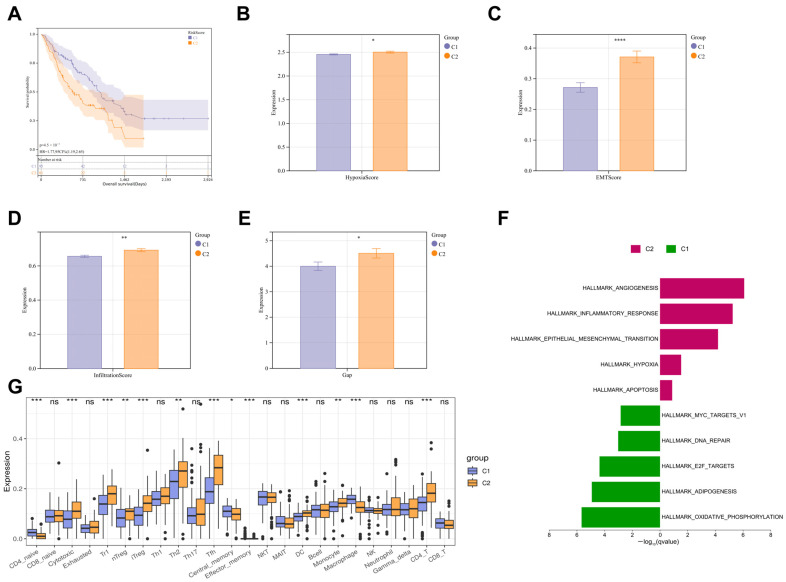
Clustering analysis based on cold-exposure-related genes (CERG). Consensus clustering of IPF patients from the GSE70866 dataset was performed based on the expression levels of CERG, resulting in the classification of IPF patients into two clusters: Cluster 1 (C1, 95 samples) and Cluster 2 (C2, 81 samples). (**A**) Kaplan–Meier (K-M) survival curves for the two clusters. (**B**–**E**) IPF-related characteristic scores for the two clusters. (**F**) GSVA analysis of the two clusters. (**G**) Immune cell infiltration profiles of the two clusters. ^ns^ *p* > 0.05, * *p* < 0.05, ** *p* < 0.01, *** *p* < 0.001, **** *p* < 0.0001.

To explore the potential association between cold exposure and IPF, we performed cluster analysis on IPF patients from the GSE70866 dataset based on the expression levels of CERGs. According to the assessment of the average silhouette width within cluster groups, the optimal number of clusters (K) was determined to be 2, at which point the average silhouette width was highest. The IPF patients were divided into two clusters: Cluster 1 (C1, 95 samples) and Cluster 2 (C2, 81 samples). PCA analysis revealed differences between the two clusters, and the expression of the majority of CERG differed significantly between them (Supplementary Figure S2). Notably, IPF patients in C2 exhibited a higher risk of mortality compared to those in C1 (Figure 1A). We further compared the IPF-related characteristic scores between the two clusters. The results demonstrated that the hypoxia, EMT, and GAP scores were significantly higher in C2 than in C1, suggesting that patients in this cluster may be experiencing more severe pathological processes (Figure 1B–E). GSVA analysis also indicated that C2 was more strongly associated with IPF-related pathways such as angiogenesis, immunity, and hypoxia (Figure 1F). Additionally, immune infiltration analysis revealed significant differences in the infiltration levels of various immune cell types between the two clusters (Figure 1G). These findings suggest that cold exposure may be a significant factor contributing to the exacerbation and poor prognosis of IPF.

### 3.2. Identification of Differentially Expressed Cold-Exposure-Related Genes in IPF

**Figure 2:**
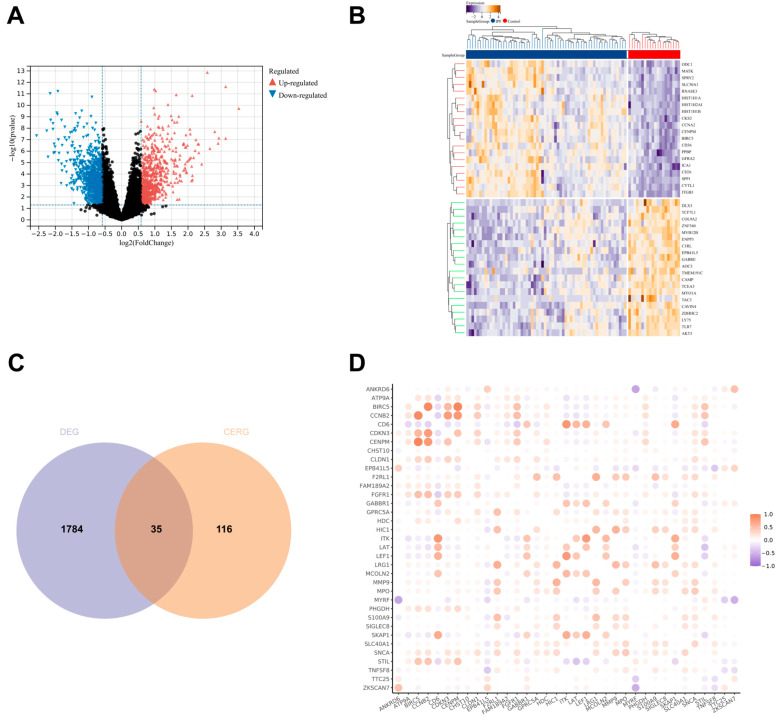
Identification of differentially expressed cold-exposure-related genes in IPF. (**A**,**B**) Differential analysis was performed on the Freiburg cohort from GSE70866, identifying 739 upregulated genes and 1080 downregulated genes (DEG) in the IPF group compared to the control group. (**C**) Venn diagram showing the intersection between DEG and CERG. (**D**) Correlation analysis of the 35 differentially expressed genes.

We further screened for CERGs that are differentially expressed in IPF. Initially, differential analysis was performed on the Freiburg cohort from the GSE70866 dataset, identifying 739 upregulated genes and 1080 downregulated genes (DEGs) in the IPF group compared to the control group (Figure 2A,B). Subsequently, these DEGs were intersected with the CERG, yielding 35 overlapping genes (Figure 2C). Correlation analysis revealed a high level of correlation in expression levels among these genes (Figure 2D).

### 3.3. Construction of a Prognostic Model Based on Differentially Expressed Cold-Exposure-Related Genes

To delve into the impact of cold-exposure-related genes on the prognosis of IPF patients, this study utilized the Freiburg cohort from the GSE70866 dataset as the training set to construct a prognostic model, with the SIENA and LEUVEN cohorts serving as validation sets, respectively.

**Figure 3:**
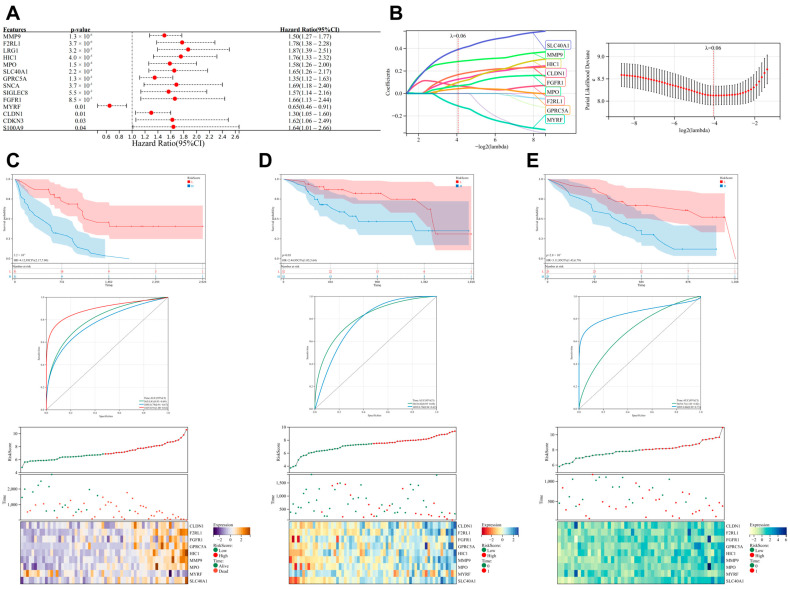
Construction and validation of a prognostic model for IPF. (**A**) Univariate Cox risk analysis of 35 cold-exposure-related differentially expressed genes in the Freiburg cohort identified 14 genes with prognostic significance. (**B**) Construction of a prognostic model using Lasso–Cox regression analysis. (**C**–**E**) Validation of the prognostic model, including K-M survival curves, ROC curves, and prognostic heatmaps. (**C**) Freiburg cohort, (**D**) SIENA cohort, and (**E**) LEUVEN cohort.

Within the Freiburg cohort, we conducted univariate risk analysis and further identified 14 genes with significant prognostic value from the previously screened 35 genes (Figure 3A). Subsequently, we employed the Lasso–Cox regression method combined with 5-fold cross-validation to optimize model parameters. With a lambda value set at 0.0764372361546994, nine key genes were ultimately determined for constructing the prognostic model (Figure 3B). The formula for the model is as follows:

RiskScore = −0.424903566503458 × ACKR3 + 0.196279622489444 × SPP1 + 0.115063964199391 × ANGPTL4 + 0.0317584621305426 × CCL2 + 0.211175047484289 × MYC − 0.219337906620058 × AXL + 0.627759546871473 × ODC1 + 0.180345676123604 × GATA1 + 0.147421726847953 × LIMS1.

We plotted the K-M survival curves and ROC curves for the prognostic model. The results demonstrated that the model exhibited significant prognostic capability in the Freiburg cohort (Figure 3C). Meanwhile, in the SIENA and LEUVEN cohorts, despite limitations due to data constraints (lack of 5-year follow-up data) precluding comprehensive validation, the model still showed a promising predictive trend (Figure 3D,E). The prognostic heatmap further visually illustrated the correlation between expression trends and prognosis.

### 3.4. Grouping Characteristics Based on RiskScore

**Figure 4:**
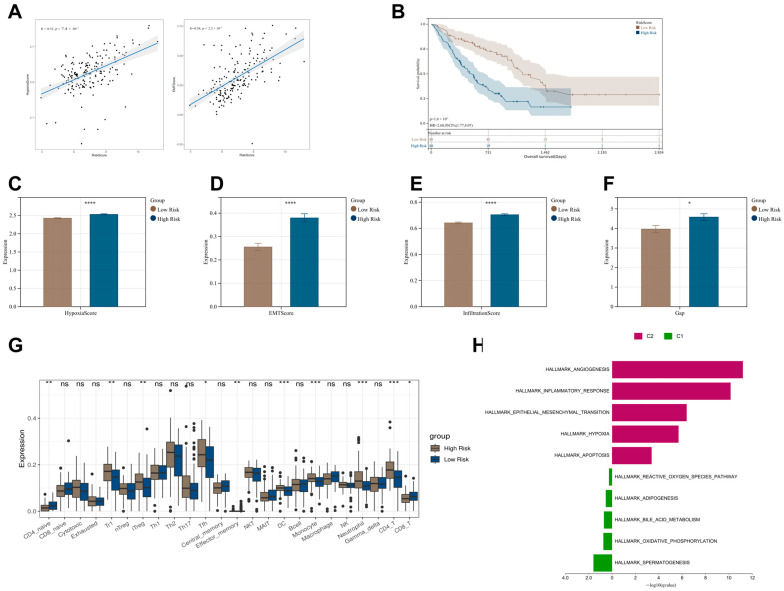
Characteristics of high-risk and low-risk IPF patient groups. (**A**) Correlation between RiskScore and Hypoxia/EMT scores. (**B**) IPF patients were divided into a high-risk group (88 samples) and a low-risk group (88 samples) based on the median RiskScore. K-M survival curves for the high-risk and low-risk groups are shown. (**C**–**F**) Scores for IPF-related characteristics in the high-risk and low-risk groups. (**G**) Immune cell infiltration in the high-risk and low-risk groups. (**H**) GSVA for the high-risk and low-risk groups. ^ns^ *p* > 0.05, * *p* < 0.05, ** *p* < 0.01, *** *p* < 0.001, **** *p* < 0.0001.

Firstly, we validated the correlation between the RiskScore from the prognostic model and IPF characteristic scores. The results indicated a positive correlation between the RiskScore and hypoxia, as well as EMT scores (Figure 4A). Subsequently, we divided all IPF patients in the GSE70866 dataset into a high-risk group (88 samples) and a low-risk group (88 samples) using the median RiskScore of the prognostic model. The K-M curve demonstrated a significantly higher mortality rate in the high-risk group compared to the low-risk group (Figure 4B). Additionally, the high-risk group exhibited higher hypoxia, EMT, immune, and GAP scores compared to the low-risk group (Figure 4C–F). Immune infiltration analysis revealed significant differences in the infiltration patterns of various immune cells between the two groups (Figure 4G). Furthermore, GSVA analysis showed that the high-risk group was closely associated with IPF-related pathways, including angiogenesis, inflammatory response, EMT, and hypoxia-signaling pathways (Figure 4H).

### 3.5. Development of a Nomogram Model

**Figure 5:**
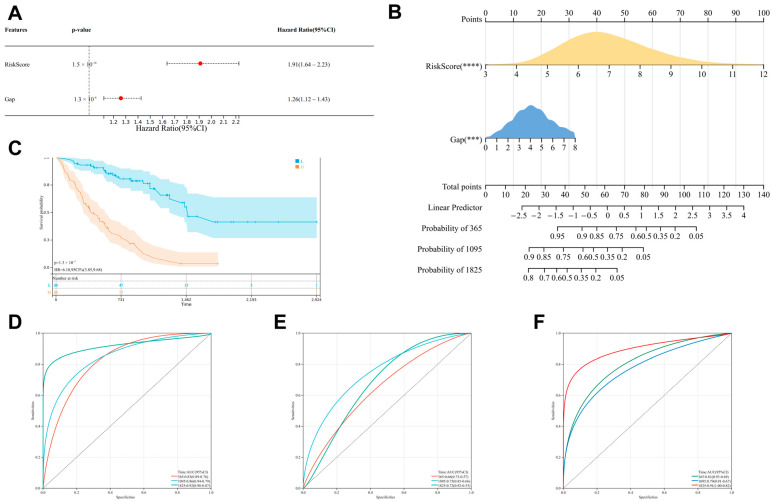
Construction of a nomogram model Based on RiskScore and GAP score. (**A**) Multivariate Cox regression analysis reveals that RiskScore and GAP are good prognostic indicators. (**B**) A nomogram model is constructed based on RiskScore and GAP score. (**C**) K-M curves demonstrate the good prognostic efficacy of the nomogram model. (**D**–**F**) ROC curves show that the prognostic performance of the nomogram is superior to that of using RiskScore or GAP score alone. Specifically, (**D**) ROC curve for the prognostic performance of the nomogram model, (**E**) ROC curve for the prognostic performance of the GAP score, and (**F**) ROC curve for the prognostic performance of the RiskScore. *** *p* < 0.001, **** *p* < 0.0001.

The GAP score is a widely recognized scoring system for assessing the severity of IPF. We constructed a nomogram model using the RiskScore and GAP score. Firstly, multivariate analysis revealed that both RiskScore and GAP are good prognostic indicators (Figure 5A). Based on this, a nomogram model was developed for IPF patients (Figure 5B). K-M survival curves and ROC curves demonstrated the good prognostic performance of the nomogram model. Moreover, the area under the ROC curve (AUC: 1 year: 0.83, 3 years: 0.86, 5 years: 0.92) was greater than that of using RiskScore alone (AUC: 1 year: 0.81, 3 years: 0.79, 5 years: 0.91) and the GAP score alone (AUC: 1 year: 0.66, 3 years: 0.75, 5 years: 0.72) (Figure 5C–F). These results indicate that the nomogram model based on RiskScore and GAP score has better prognostic capability.

### 3.6. Identification of Differentially Expressed Genes Between High-Risk and Low-Risk Groups

**Figure 6:**
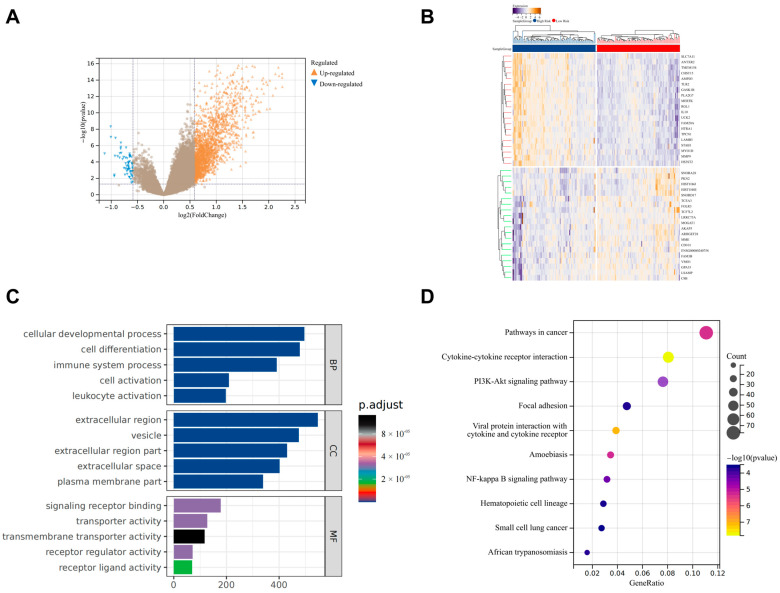
Differential expression analysis and functional enrichment analysis between high-risk and low-risk Groups. (**A**,**B**) A total of 1764 upregulated genes and 59 downregulated genes were identified in the high-risk group compared to the low-risk group. (**C**) GO analysis of the differentially expressed genes. (**D**) KEGG analysis of the differentially expressed genes.

We further conducted differential expression analysis between the high-risk and low-risk groups, identifying 1764 upregulated genes and 59 downregulated genes (Figure 6A,B). GO analysis revealed that these genes are primarily enriched in biological processes related to cell development, activation, differentiation, and the immune system (Figure 6C). KEGG analysis showed that they are associated with inflammatory-related-signaling pathways such as PI3K-Akt and NF-κB, as well as cancer-signaling pathways (Figure 6D). This finding not only reiterates the central role of immunity and inflammation in the pathogenesis of IPF but also suggests a potential link between cold exposure and immunity.

### 3.7. Identification of Genes Associated with the High-Risk Group Using WGCNA

**Figure 7:**
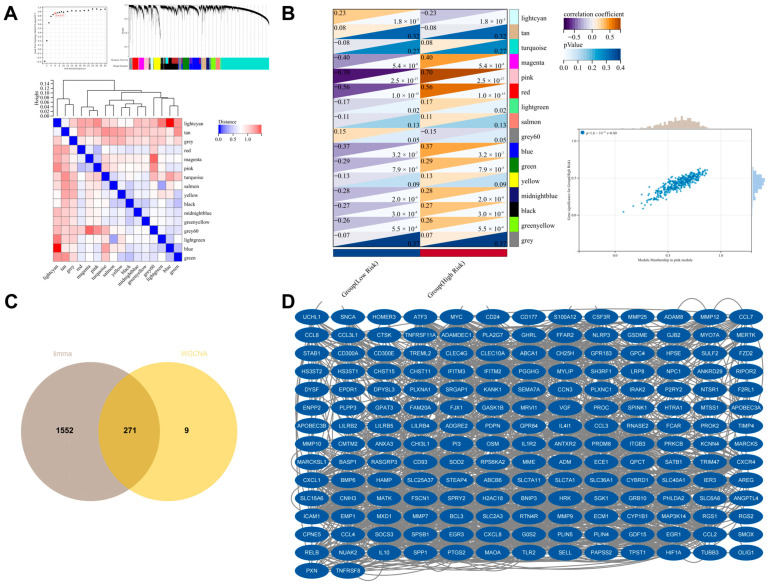
Identification of genes associated with high risk (HRRG). (**A**,**B**) WGCNA was performed on IPF patients from the GSE70866 dataset, resulting in the identification of 16 modules. The pink module was found to be the most correlated with the high-risk group in IPF, and 280 genes were screened from this module. (**C**) Venn diagram showing the intersection between differentially expressed genes from high-risk and low-risk groups and the module genes identified by WGCNA. (**D**) Through PPI analysis, 184 genes associated with HRRG were identified.

We further screened genes associated with the high-risk group using WGCNA. In the IPF patient samples from the GSE70866 dataset, we systematically constructed a gene co-expression network and successfully identified 16 modules with significant co-expression characteristics (Figure 7A). Among these, the pink module exhibited the strongest correlation with the high-risk status (Figure 7B), and a total of 280 genes were screened from this module. Finally, by intersecting these genes with the differentially expressed genes, we obtained 271 genes (Figure 7C). Through PPI analysis, we ultimately determined 184 genes associated with high risk in IPF (HRRG) (Figure 7D).

### 3.8. Selection of Signature Genes

**Figure 8:**
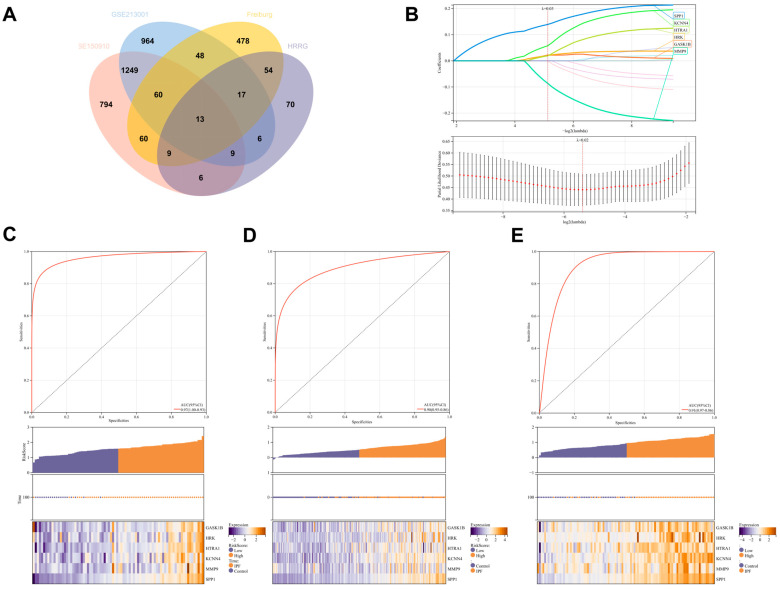
Selection of signature genes. (**A**) Differential expression analysis was conducted on the Freiburg cohort from GSE70866, as well as on GSE150910 and GSE213001. By intersecting the commonly differentially expressed genes from these three datasets with the 184 HRRG, we ultimately obtained 13 genes. (**B**) Lasso–Cox regression identified six signature genes: *GASK1B*, *HRK1*, *HTRA1*, *KCNN4*, *MMP9,* and *SPP1*. (**C**–**E**) ROC curves and heatmaps demonstrate the diagnostic efficacy and expression trends of the signature genes. Panel **C** represents the Freiburg cohort, Panel **D** represents GSE150910, and Panel **E** represents GSE213001.

To further screen for signature genes, we conducted differential expression analysis on the Freiburg cohort from GSE70866, as well as on GSE150910 and GSE213001. By intersecting the commonly differentially expressed genes from these three datasets with the 184 HRRG, we ultimately obtained 13 genes (Figure 8A). Using Lasso–Cox regression analysis with 5-fold cross-validation, we finally identified six signature genes: *GASK1B*, *HRK1*, *HTRA1*, *KCNN4*, *MMP9*, and *SPP1*. ROC curves and heatmaps demonstrate the diagnostic efficacy of the combination of these signature genes in the Freiburg cohort, GSE150910, and GSE213001 (Figure 8C–E).

### 3.9. Validation and Functional Analysis of Signature Genes

**Figure 9:**
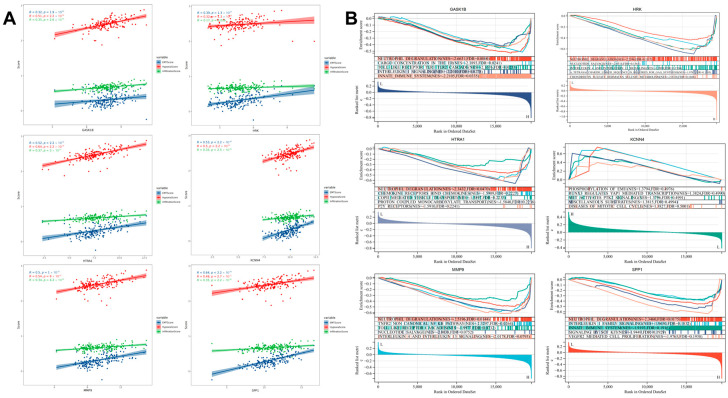
Correlation analysis and GSEA analysis of signature genes with IPF characteristic scores. (**A**) The six signature genes exhibit positive correlations with hypoxia score, EMT score, and immune score. (**B**) Single-gene GSEA analysis of the six genes.

**Figure 10:**
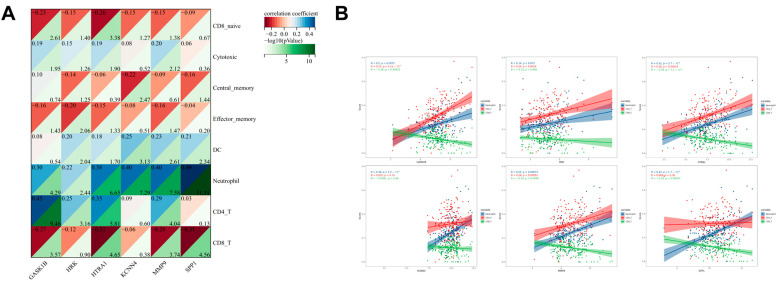
Correlation analysis of signature genes with immune cell infiltration. (**A**) Relationship between the signature genes and the levels of immune cell infiltration in IPF patients. (**B**) Association of the signature genes with CD4^+^ T cells, CD8^+^ T cells, and neutrophils.

We further validated the roles of the signature genes in IPF. Correlation analysis revealed that the signature genes exhibited significant positive correlations with the hypoxia score, EMT score, and immune score (Figure 9A). Single-gene GSEA analysis showed that, except for *KCNN4*, the other five genes were significantly associated with the neutrophil degranulation process, highlighting their potential roles in regulating the neutrophil function and inflammatory response. Additionally, GSEA analysis revealed significant associations between these genes and multiple immune system-related or inflammation-related pathways, including the interleukin-signaling pathway, chemokine-signaling pathway, and Toll-like receptor-signaling pathway (Figure 9B). We further explored the relationship between the signature genes and immune cell infiltration and found that they were correlated with the infiltration levels of multiple immune cells, particularly CD4^+^ T cells, CD8^+^ T cells, and neutrophils (Figure 10A,B).

## 4. Discussion

This study explored the potential link between cold exposure and IPF. Due to the lack of human-specific data, the CERGs in this study were derived from mouse RNA-Seq data. Numerous studies have demonstrated the stability and feasibility of using cold-exposed mouse models [39,40,41,42]. Moreover, evidence suggests that cold exposure can exacerbate inflammatory responses through various pathways, leading to increased lung damage from various causes [43,44,45,46]. In this study, we innovatively performed clustering analysis on IPF patients based on cold exposure and successfully identified subgroups of IPF patients with different severities. This finding not only reveals the potential value of cold-exposure-related genes in classifying IPF patients but also provides a new perspective for understanding the heterogeneity of IPF. Further analysis showed that IPF patients exhibited distinct patterns of immune cell infiltration in both the subgroups identified by clustering based on cold-exposure-related gene expression and the high-risk and low-risk groups classified according to the prognostic model. This finding suggests that cold exposure may be involved in the pathological process of IPF by influencing the infiltration and activation status of immune cells. However, this hypothesis may require further experimental validation.

Based on cold-exposure-related genes, we established a stable prognostic model. The construction and validation process of this model suggests that cold-exposure-related genes may serve as important biomarkers for predicting patient prognosis. The GAP (Gender, Age, Physiology) scoring system is a tool used to assess the severity of illness and prognostic risk in IPF patients, incorporating patient gender, age, and two pulmonary function parameters (FVC and DLco). It enables the rapid assessment of prognostic risk and demonstrates high accuracy in predicting survival rates among IPF patients [34]. However, it has certain limitations; for instance, it primarily relies on static patient data for evaluation without fully considering the dynamic changes in these parameters over the course of the disease. The accuracy of the GAP scoring system may vary across different populations [47,48]. In light of this, numerous studies are currently dedicated to exploring new prognostic indicators, aiming to enhance the accuracy of prognostic assessments when used in conjunction with the GAP scoring system [49,50,51]. Studies have shown that combining circulating biomarker levels with the GAP score significantly improves predictive ability compared to using the GAP score alone [52]. In our study, we found that the prognostic model based on cold-exposure-related genes outperformed the GAP score in prognostic capability. Furthermore, the nomogram model constructed using the RiskScore from our prognostic model and the GAP demonstrated even better prognostic ability.

We further screened six IPF high-risk signature genes related to cold exposure: *GASK1B*, *HRK1*, *HTRA1*, *KCNN4*, *MMP9*, and *SPP1*. Previous studies have demonstrated that *SPP1*, *MMP9*, and *KCNN4* play a role in promoting fibrosis in the progression of IPF. Specifically, in IPF patients, *SPP1*, which is highly expressed in alveolar epithelial cells, can induce the proliferation and migration of fibroblasts and epithelial cells, thereby accelerating the process of pulmonary fibrosis. Furthermore, lung macrophages with high *SPP1* expression constitute an important profibrotic macrophage subpopulation that plays a crucial role in lung tissue repair and fibrosis [53,54]. *MMP9* is primarily involved in the degradation and remodeling of extracellular matrix components. *MMP9* expressed by activated neutrophils is considered one of the important pathogenic mechanisms of UIP [55]. *MMP9* expressed by alveolar macrophages contributes to the remodeling of the pulmonary basement membrane, basement membrane stripping, and structural remodeling within the interstitium and alveoli, thereby promoting the development of pulmonary fibrosis [56]. *KCNN4* promotes Smad2/3 signaling in IPF-derived fibroblasts, facilitating the differentiation of fibroblasts into myofibroblasts and promoting the formation of pulmonary fibrosis [57]. In our study, the increased expression of *SPP1*, *MMP9*, and *KCNN4* in the high-risk IPF group is consistent with previous findings, further confirming the significant role of these genes in the progression of IPF.

In this study, the expression levels of *GASK1B*, *HRK1*, and *HTRA1* were all elevated in the high-risk IPF group. However, the specific mechanisms of their roles in IPF have not been reported in previous studies. *GASK1B*, also known as *FAM198B*, has the function of targeting the SMAD2-signaling pathway and can regulate the polarization of macrophages towards the M2 phenotype. As a classic profibrotic cell subtype, M2 macrophages play an important role in the onset and progression of IPF [58,59,60]. Moreover, *GASK1B* may functionally be related to IPF-associated pathological features such as angiogenesis and EMT [61]. *HRK1* is a K+ channel-related gene that regulates the proliferation of various cells, including glial cells, microglia, and fibroblasts [62,63,64,65]. Although there is currently no direct evidence indicating an interaction between *HRK1* and IPF, its homolog protein KCNJ2 is significantly increased in the fibroblasts and bronchoalveolar lavage fluid of IPF patients and has been used as a biomarker for the differential diagnosis of IPF and other interstitial lung diseases [66]. *HTRA1* is a highly conserved serine protease that promotes the transport and secretion of type I collagen from the endoplasmic reticulum to the Golgi apparatus and activates TGF-β signaling, thereby promoting the fibrosis process [67,68]. In summary, combined with the results of this study, we speculate that *GASK1B*, *HRK1,* and *HTRA1* may play important roles in the pathogenesis of IPF.

This study also has certain limitations and shortcomings. Firstly, due to the lack of samples from IPF patients exposed to cold conditions, we had to rely on mouse transcriptome sequencing results to identify CERG. However, the limited sample size and interspecies differences may have an impact on the conclusions of this study, leading to certain limitations in the research findings. Secondly, this study primarily employed bioinformatics methods for indirect analysis, revealing potential relationships between cold exposure and IPF, but direct clinical evidence is still lacking. In the future, it may be necessary to collect samples from IPF patients from different regions to further validate and explore this relationship. Lastly, this study identified six high-risk signature genes related to cold exposure in IPF through analysis, but these have not been experimentally validated in tissue specimens. Therefore, the specific mechanisms of action of these genes in IPF need to be further explored through clinical samples, animal experiments, and other means.

## 5. Conclusions

Cold exposure may be a significant environmental factor mediating the progression of IPF. A prognostic model based on cold-exposure-related genes could serve as a promising indicator for the prognosis of IPF. *GASK1B*, *HRK1*, *HTRA1*, *KCNN4*, *MMP9*, and *SPP1* may represent potential biomarkers and therapeutic targets for IPF.

## Figures and Tables

**Table 1:** Detailed information on the dataset.

Data	Species	GPL	Data Type	Information
GSE183321	Mus musculus	-	RNA-Seq	Four cold-exposure and five control group samples
GSE70866	Homo sapiens	GPL14550 GPL17077	Array	Freiburg queue: 62 IPF and 20 control samples SIENA queue: 50 IPF samples LEUVEN queue: 64 IPF samples
GSE150910	Homo sapiens	-	RNA-Seq	103 IPF and 103 Control samples
GSE213001	Homo sapiens	-	RNA-Seq	62 IPF and 41 Control samples

## Data Availability

All data can be obtained from the corresponding author.

## Supplementary Materials

The following supporting information can be downloaded at: https://www.mdpi.com/article/10.3390/biomedicines13030690/s1, Figure S1: Differential Analysis and Functional Enrichment Analysis of GSE183321 (Cold Exposure Group and Control Group); Figure S2: Cluster Analysis Based on Cold Exposure-Related Genes (CERG).
