# Supplementary material for: Developing an IPF Prognostic Model and Screening for Key Genes Based on Cold Exposure-Related Genes Using Bioinformatics Approaches
Source: Biomedicines. 2025 Mar 11;13(3):690. doi: 10.3390/biomedicines13030690 (PMC11940207; doi:10.3390/biomedicines13030690)
Supplement: Supplementary file 1 [file biomedicines-13-00690-s001.zip › biomedicines-3464614-supplementary.pdf]

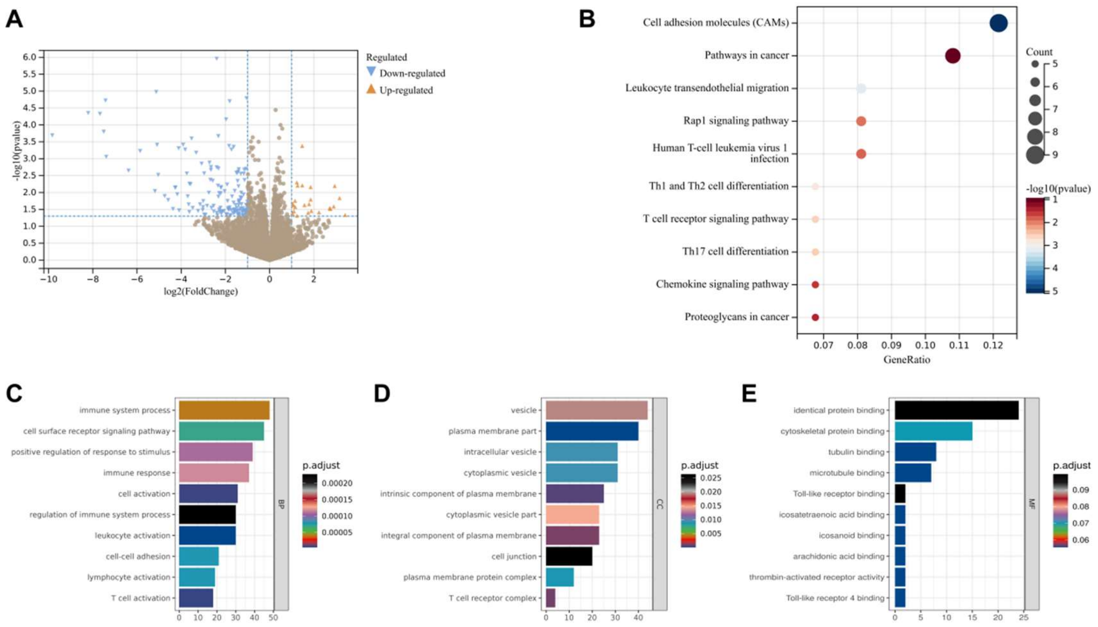

Supplementary Figure S1. Differential Analysis and Functional Enrichment Analysis of GSE183321 (Cold Exposure Group and Control Group)

(A) Volcano plot of differentially expressed genes. (B) KEGG analysis of differentially expressed genes. (C-E) GO analysis of differentially expressed genes.

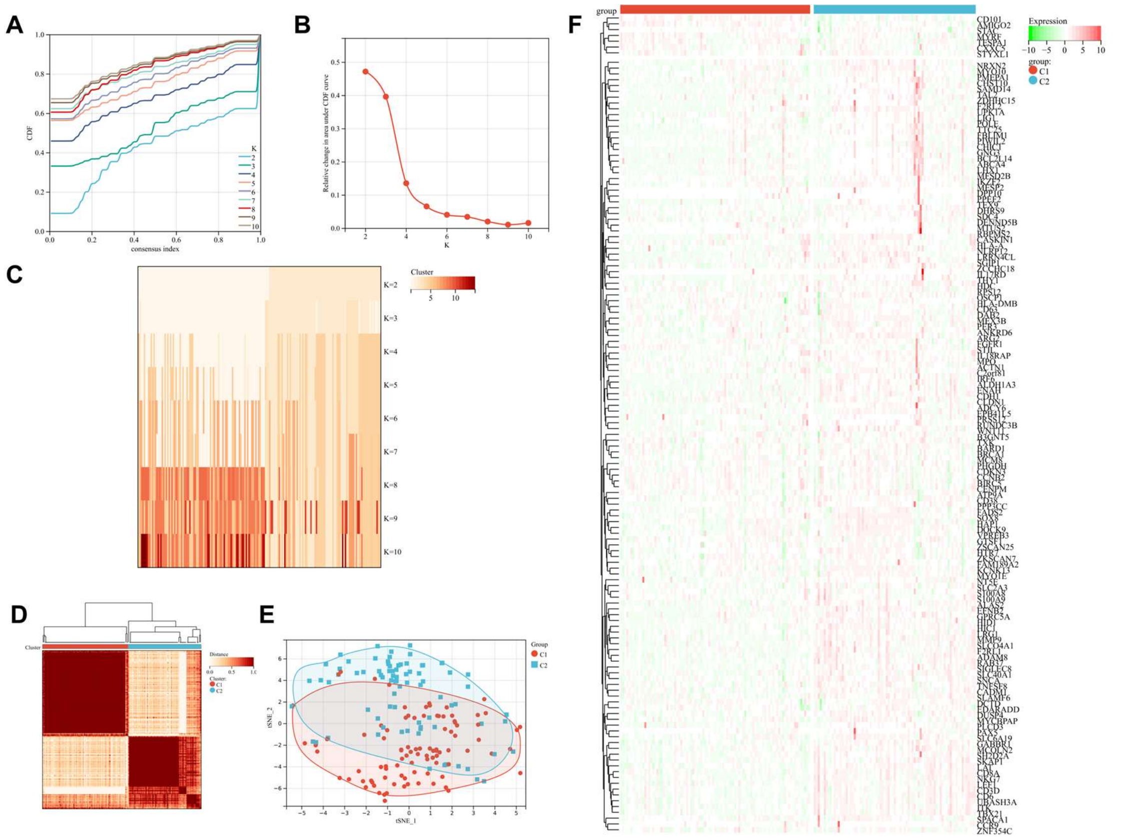

Supplementary Figure S2. Cluster Analysis Based on Cold Exposure-Related Genes (CERG)

(A) Cumulative distribution function (CDF) plot. (B) Area under the CDF curve. (C) Silhouette width of sample clustering. (D) Heatmap of clustering results. (E) PCA analysis of the two clusters. (F) Expression levels of CERG in the two clusters.
